# 

**Published:** 1901-12-14

**Authors:** 


### NOTICE TO CORRESPONDENTS.

All MSS., Letters, Books for Review, and other matters intended for the Editor should be addressed The Editor, "The Hospital," The Hospital Building, 28 & 29 Southampton Street, Strand, W.C.

The Editor cannot undertake to return rejected MS., even when accompanied by stamped directed envelope.

**Notice to Advertisers and Subscribers.**

All Advertisements, Orders for Copies of The Hospital, and Business Communications should be addressed to **THE MANAGER (Not to the Editor),** The Hospital, 28 & 29 Southampton Street, Strand, London, W.C.

The Cost of Subscription, post free, is as follows:—Per week, 2½d.; per quarter, 2s. 8d.; per half-year, 5s. 3d.; per annum, 10s. 6d. Foreign:—Per annum, 15s. 2d., payable, in all cases, in advance.

**NOTICE.—Vol. XXX. of "The Hospital" (April 1901, to September 1901), in handsome cloth binding can now be obtained at the office of this Journal, price, 7s. 6d. Cases for binding the Numbers contained in this Volume 1s. 6d. Index to Vol. XXX., 2½d., post free.**

**The Monthly Part for December is now ready. Price 6d., by post 8d.**

### Scraps and Gleanings.

The whole cost of the new Women and Children's Hospital, at Leeds, which is now being erected is estimated at about £24,000, of which sum £6,000 more remains to be collected, and without this sum the building cannot be properly equipped.

A fund is being collected to endow a bed in the New Hospital for Women, Euston Road, for patients suffering from cancer. This endowed bed is to be a memorial to the late Emperor and Empress Frederic. Messrs. Barclay, of the Pall Mall East, are the bankers of the fund.

A sum of upwards of £7,500 was collected at the recent festival dinner in aid of the building fund of the Victoria Hospital for Children at which Lord Roberts presided. The estimated cost of the new hospital is £30,000, and of that sum £6,000 had been collected before the dinner.

There is a deficiency of nearly £950 on the Special Building Fund of the Royal Albert Hospital, Devonport, and the committee appeal for further donations and subscriptions. The total amount charged to this fund for structural alterations is £6,820, which includes the expenditure on the new nursing home, the re-drainage of the whole institution, etc.

### The Student of Medicine.

### Appointments.

**Charles C. Choyce**, M.B., Ch.B.Edin., B.Sc.N.Z., appointed House Physician to the Leicester Infirmary. **John Cunningham**, M.B., C.M., appointed Certifying Factory Surgeon for the Stewarton District of North Ayrshire. **J. P. Dee**, M.B., B.S.R.U.I., appointed District Medical Officer of the Tendring Union. **William Ireland Donaldson**, B.A., M.D.Dub., appointed Medical Superintendent to the London County Asylum, The Manor, Epsom. **Andrew Duncan**, M.D., B.S.Lond., M.R.C.P., F.R.C.S., appointed Physician to the Westminster Dispensary. **Alfred Hewetson**, M.R.C.S.Eng., L.R.C.P.Lond., appointed Civil Surgeon of the Military Depôt, Fremling Green, Aldershot. **A. W. Limont**, M.B., M.S Edin., appointed District Medical Officer of the Truro Union. **V. Warren Low**, F.R.C.S., appointed Surgeon to Out-patients at the Great Northern Central Hospital. **F. E. Nichol**, M.B., B.C.Cantab., appointed Certifying Factory Surgeon for the Margate District of Kent. **Leonard A. Parry**, F.R.C.S.Eng., B.S., M.D.Lond., appointed Assistant Surgeon to the Royal Alexandra Hospital for Children, Brighton. **H. Fraser Stokes**, M.D., appointed Medical Officer to the Highbury Truant School of the London School Board. **W. Greenwood Sutcliffe**, F.R.C.S., appointed Surgeon to the Royal Sea Bathing Hospital. **Walter Essex Wynter**, M.D., B.S., F.R.C.S., F.R.C.P., elected Physician to the Middlesex Hospital.

